# Arecoline-induced myofibroblast transdifferentiation from human buccal mucosal fibroblasts is mediated by ZEB1

**DOI:** 10.1111/jcmm.12219

**Published:** 2014-01-08

**Authors:** Yu-Chao Chang, Chung-Hung Tsai, You-Liang Lai, Cheng-Chia Yu, Wan-Yu Chi, Jung Jung Li, Wen-Wei Chang

**Affiliations:** a School of Dentistry, Chung Shan Medical UniversityTaichung, Taiwan; b Department of Dentistry, Chung Shan Medical University HospitalTaichung, Taiwan; c Department of Pathology, Chung Shan Medical University HospitalTaichung, Taiwan; d Institute of Medicine, Chung Shan Medical UniversityTaichung, Taiwan; e School of Biomedical Sciences, Chung Shan Medical UniversityTaichung, Taiwan; f Institute of Oral Science, Chung Shan Medical UniversityTaichung, Taiwan; g Department of Medical Research, Chung Shan Medical University HospitalTaichung, Taiwan

**Keywords:** arecoline, myofibroblasts, buccal mucosal fibroblasts, ZEB1, oral submucous fibrosis, α-SMA

## Abstract

Oral submucous fibrosis (OSF) is considered as a pre-cancerous condition of the oral mucosa and is highly associated with habitual areca quid chewing. Arecoline is the major alkaloid in areca quid and is thought to be involved in the pathogenesis of OSF. Our previous studies have demonstrated that arecoline could induce epithelial–mesenchymal transition (EMT)-related factors in primary human buccal mucosal fibroblasts (BMFs). Therefore, we investigated the expression of zinc finger E-box binding homeobox 1 (ZEB1), which is a well-known transcriptional factor in EMT, in OSF tissues and its role in arecoline-induced myofibroblast transdifferentiation from BMFs. The expression of ZEB1, as well as the myofibroblast marker α-smooth muscle actin (α-SMA), was significantly increased in OSF tissues, respectively. With immunofluorescence analysis, arecoline induced the formation of α-SMA-positive stress fibres in BMFs expressing nuclear ZEB1. Arecoline also induced collagen contraction of BMFs *in vitro*. By chromatin immunoprecipitation, the binding of ZEB1 to the α-SMA promoter in BMFs was increased by arecoline. The promoter activity of α-SMA in BMFs was also induced by arecoline, while knockdown of ZEB1 abolished arecoline-induced α-SMA promoter activity and collagen contraction of BMFs. Long-term exposure of BMFs to arecoline induced the expression of fibrogenic genes and ZEB1. Silencing of ZEB1 in fibrotic BMFs from an OSF patient also suppressed the expression of α-SMA and myofibroblast activity. Inhibition of insulin-like growth factor receptor-1 could suppress arecoline-induced ZEB1 activation in BMFs. Our data suggest that ZEB1 may participate in the pathogenesis of areca quid–associated OSF by activating the α-SMA promoter and inducing myofibroblast transdifferentiation from BMFs.

## Introduction

Oral submucous fibrosis (OSF) is a chronic progressive scarring disease that is characterized by the submucosal accumulation of dense fibrous connective tissue with inflammatory cell infiltration and epithelial atrophy and is considered a pre-cancerous condition of the oral mucosa 1. Based on epidemiological evidence, OSF is highly associated with the areca quid chewing habit 2; however, the mechanism by which areca nuts affect the oral mucosa is not fully understood. In studies of OSF pathogenesis, the alteration of extracellular matrix (ECM) components has been widely investigated. Increased collagen synthesis or decreased collagen degradation may play a role in the development of OSF 3. Inflammation also plays a role in the development of OSF. Inflammatory cytokines [tumour necrosis factor-α, interleukin (IL)-6] or mediators (cyclooxygenase-2, prostaglandin) secreted by oral keratinocytes may contribute to the oral fibrogenic condition 1. Recently, one report demonstrated that arecoline, the major alkaloid in areca quid, can activate integrin αvβ6 expression in oral keratinocytes to activate transforming growth factor (TGF)-β1 and further induce the transdifferentiation of oral fibroblasts into myofibroblasts 4.

During wound healing and organ fibrosis, myofibroblasts are the major cell type that secretes collagen and reorganizes the ECM 5. Deregulation of myofibroblast activity has been found in fibrosis of several organs, including the liver, heart and lung 6. The typical molecular feature of differentiated myofibroblasts is the expression of alpha smooth muscle actin (α-SMA) and fibronectin 5. The local fibroblasts within tissues are considered the predominant source of myofibroblasts, although myofibroblasts can also be contributed from other cell types, such as endothelial cells and epithelial cells 6. From our previous studies, we demonstrated that up-regulation of several molecules involved in the epithelial–mesenchymal transition (EMT), such as plasminogen activator inhibitor-1 7, insulin-like growth factor-1 (IGF-1) 8 and NF-κB 9, was expressed in human buccal mucosal fibroblasts (BMFs) after arecoline treatment. In addition, arecoline could also induce the expression of vimentin 10 in human BMFs. These findings suggest that the EMT programme may be directly involved in the pathogenesis of OSF.

Zinc finger E-box binding homeobox 1 (ZEB1) is a well-known factor in the activation of the EMT programme. ZEB1 functions as a transcription repressor to negatively regulate the expression of polarity markers, such as E-cadherin, MucI and Pkp3 11. In addition to the role of transcription repressor, ZEB1 also activates gene expression. Zinc finger E-box binding homeobox 1 activated ATPase1 in Madin-Darby canine kidney (MDCK) cells, but repressed ATPase1 in rat fibroblasts 12, which indicates that ZEB1 might have cell type–specific functions. In the mouse model of renal fibrosis, ZEB1, which is a target of microRNA 192 (miR-192), was found to repress the expression of collagen 13. Inhibition of miR-192 increased ZEB1 and decreased collagen, inhibiting renal fibrosis 13. Zinc finger E-box binding homeobox 1 was also observed in cancer fibroblasts from human colon cancer, although its role in cancer fibroblasts remains unclear 14. The role of ZEB1 in areca nut chewing–associated OSF also remains unknown.

In this study, we first demonstrate that ZEB1 expression is significantly increased in areca nut chewing–associated OSFs. Arecoline, the major alkaloid in areca quid, induced α-SMA, as well as ZEB1 expression, in BMFs. We further demonstrate that ZEB1 binds to the E-box region in the α-SMA promoter to activate its activity. Knockdown of ZEB1 abolished arecoline-induced α-SMA promoter activity, protein expression and collagen gel contraction in BMFs. We also demonstrate that arecoline could up-regulate the expression of insulin-like growth factor receptor-1 (IGF-1R) in BMFs and suppression of the activation of IGF-1R could inhibit the arecoline-induced ZEB1 activation. Our data suggest that arecoline-induced ZEB1 expression is involved in the pathogenesis of OSF.

## Materials and methods

### Reagents and antibodies

Arecoline was purchased from Sigma–Aldrich (St. Louis, MO, USA). *N*-acetyl cysteine (NAC) and (picropodophyllin) PPP were purchased from Tocris Bioscience (Bristol, UK). Monoclonal mouse anti-human antibodies against α-SMA (1A4), vimentin (9E7E7), and ZEB1 (416A7H10) and rabbit polyclonal anti-human antibody against COL1A1 (H-197) and IGF-1Rβ (C-20) were purchased from Santa Cruz Biotechnology Inc. (Santa Cruz, CA, USA). Rabbit polyclonal anti-human antibodies against p-IGF-1R^Tyr1161^ and Glyceraldehyde 3-phosphate dehydrogenase (GAPDH) antibody was purchased from GeneTex Inc. (Hsinchu City, Taiwan). A collagen solution from bovine skin was purchased from Sigma–Aldrich. Horseradish peroxidase–conjugated antimouse IgG or anti-rabbit IgG antibodies were purchased from PerkinElmer (Waltham, MA, USA). Alexa 488–conjugated antimouse IgG and Alexa 594–conjugated anti-rabbit IgG antibodies were purchased from Jackson ImmunoResearch Laboratories Inc. (West Grove, PA, USA).

### Immunohistochemistry and immunofluorescence

For immunohistochemistry, formalin-fixed, paraffin-embedded specimens of 10 normal buccal mucosa from non-areca quid chewers and 30 OSF specimens from areca quid chewers were collected in the Department of Dentistry, Chung Shan Medical University Hospital with a protocol approved by the Institutional Review Board at the Chung Shan Medical University Hospital. Diagnosis was based on histological examination of haematoxylin–eosin-stained sections. Five micron sections were stained with the monoclonal anti-ZEB1 or anti-α-SMA antibody (1:100 dilution) using a standard avidin–biotin–peroxidase complex method. 3,3′-Diaminobenzidine (DAKO, Carpinteria, CA, USA) was then used to detect the antibody binding. Negative controls included serial sections from which either the primary or secondary antibodies were excluded. The preparations were counterstained with haematoxylin, mounted with Permount (Merck, Darmstadt, Germany) and examined by light microscopy. For immunofluorescence, 1 × 10^4^ BMFs/well were seeded into wells of a μ-slide (ibidi GmbH, Martinsried, Germany). After fixing with 3.7% formaldehyde in PBS, wells were blocked with 1% BSA in PBS at room temperature for 1 hr and incubated with primary antibodies (1:100 dilution) at 4°C overnight. After washing with 0.1% Tween-20 in PBS, the wells were then incubated with fluorescent-conjugated secondary antibodies (1:100 dilution) at room temperature for 1 hr. Cells were further counterstained with 0.1 μg/ml DAPI (2-(4-Amidinophenyl)-6-indolecarbamidine dihydrochloride), and the fluorescence signals were observed with an inverted fluorescence microscope (Motic Group Co. Ltd., Xiamen, China).

### Cell culture

Three healthy individuals without an areca quid chewing habit and one OSF patient with an areca quid chewing habit who visited the Oral Medicine Center (Chung Shan Medical University Hospital, Taichung, Taiwan) were enrolled in this study with informed consent and the protocol was approved by Institutional Review Board of Chung Shan Medical University Hospital. Biopsy specimens were derived from histologically normal oral mucosa at the time of surgical third molar extraction. Fibroblast cultures were grown and maintained using the explant method as described previously 15. Cell cultures between the third and eighth passages were used in this study.

### Western blot analysis

Cells were lysed with NP-40 lysis buffer, and protein concentration was determined by BCA protein assay reagent (Thermo Fisher Scientific Inc., Rockford, IL, USA). A total of 25 μg of total protein were separated by SDS-PAGE and transferred to a polyvinylidene difluoride (PVDF) membrane (Millipore, Billerca, MA, USA). Protein detection was conducted by the SignalBoost™ Immunodetection Enhancer kit (Calbiochem, San Diego, CA, USA) according to the manufacturer's recommendation. Briefly, the primary antibody was diluted with a primary antibody solution and incubated with the PVDF membrane at 4°C overnight. After washing with 0.1% Tween-20 in a Tris buffer solution, the membrane was incubated for 1 hr at room temperature with a secondary antibody that was diluted with secondary antibody buffer. The signals were developed using an ECL-plus chemiluminescence substrate (Perkin-Elmer, Waltham, MA, USA) and captured using a LAS-1000plus Luminescent Image Analyzer (GE Healthcare Biosciences, Piscataway, NJ, USA). The band intensity was quantified using Bio1D software (Vilber Lourmat, Marne-la-Vallée, France).

### Collagen contraction assay

Buccal mucosal fibroblasts (2 × 10^5^) were suspended in 0.5 ml of a 2 mg/ml collagen solution (Sigma–Aldrich) and added into one well of a 24-well plate. The plate was incubated at 37°C for 2 hrs, which caused polymerization of the collagen gels. After detaching the gels from the wells, the gels were further incubated in 0.5 ml minimum essential medium eagle-α medium with or without arecoline for 48 hrs. The contraction of the gels was evaluated by photographing the gels and using ImageJ software (National Institutes of Health, Bethesda, MA, USA) to calculate the gel area after contraction.

### Luciferase-based reporter assay

The full-length α-SMA promoter was amplified from genomic DNA of immortalized human gingival keratinocytes (SG cells) and cloned into the pGL3-basic luciferase reporter vector (Promega, Madison, WI, USA) using the restriction enzymes *SacI* and *BglII* and the following primers: forward: 5′-CACTTGGAGCTCTCTGCTAAATTGCTCGGTGAC-3′ and reverse: 5′-CAAGTGAGATCTATTTTACAGAAGACATGCAT-3′. The assay was conducted using a dual reporter assay system. Briefly, the α-SMA promoter–containing vector (pGL3-SMAP) was cotransfected with a reference Renilla luciferase vector (Promega) at a ratio of 10:1. After transfection for 48 hrs, cells were lysed in passive lysis buffer (PJK GmbH, Kleinblittersdorf, Germany). Luciferase activity was detected with Beetle-Juice [for firefly luciferase (FLuc)] and Gaussia-Juice [for Renilla luciferase (RLuc)] substrates (PJK GmbH), and luminescence was counted on a luminescence reader (Promega). The results of the FLuc count were normalized to RLuc, which represented the transfection efficiency of each sample.

### Lentivirus-based shRNA delivery

The lentiviral vectors carrying LacZ-specific shRNA (sh-LacZ, TRCN0000231722) or ZEB1-specific shRNA (sh-ZEB1(1): TRCN0000017565; sh-ZEB1(2): TRCN0000017567; sh-ZEB1(3): TRCN0000369267) were obtained from the National RNAi Core Facility at the Institute of Molecular Biology (Academia Sinica, Taipei, Taiwan), and shRNA lentiviruses were produced by the RNAi core at the Research Center of Clinical Medicine, National Cheng Kung University Hospital (Tainan, Taiwan). For virus transduction, cells were plated at 2 × 10^5^ cells per well in six-well plates and transiently transduced with lentivirus (MOI = 1) in the presence of 8 μg/ml polybrene (Sigma–Aldrich) for 24 hrs. The cells then were selected with 2 μg/ml puromycin (Sigma–Aldrich) and were harvested at 96 hrs after transduction for subsequent analyses.

### Chromatin immunoprecipitation

Cells were harvested by trypsin-Ethylenediaminetetraacetic acid (EDTA) and fixed with 1% formaldehyde at room temperature for 10 min. After quenching the formaldehyde with 125 mM glycine, the cells were lysed with mammalian cell lysis buffer (Pierce, Thermo Fisher Scientific Inc.) and treated with micrococcal nuclease (Fermentas, Thermo Fisher Scientific Inc.) at 37°C for 20 min. The fragmented DNA solution was further diluted with ChIP dilution buffer (0.01% SDS, 1.1% Triton X-100, 1.2 mM EDTA, 16.7 mM Tris-HCl and 167 mM NaCl) and pre-cleared by incubation with 10 μl Protein A Mag Sepharose (GE Healthcare) at room temperature for 2 hrs. After the pre-clear step, 1 μg of anti-ZEB1 antibody or normal mouse IgG was added to the cell lysate, and the mixture was incubated at 4°C overnight. After washing, protein/DNA complexes were eluted using elution buffer (1% SDS and 100 mM NaHCO_3_). Reverse crosslinking of the protein/DNA complexes was performed by treating with 200 mM NaCl at 65°C for at least 5 hrs, and the proteins were digested with proteinase K. The DNA was further purified using a PCR cleanup kit (Qiagen, Hilden, Germany). The E-box region of the α-SMA promoter was detected using the following primer pair: forward: 5′-CTGCCCATTACCCTAGCTCA-3′ and reverse: 5′-CCACTGGTCTGCTCATGAAA-3′. The PCR was performed by a thermal cycler (Bio-Rad, Hercules, CA, USA) with 35 cycles of 94°C for 30 sec., 58°C for 1 min. and 72°C for 1 min. PCR products were analysed with 2% agarose gel and the intensities of bands were quantified with Bio1D software.

### Quantitative real-time RT-PCR

Total RNA was extracted using a Quick RNA MiniPrep kit (Zymo Research, Irvine, CA, USA) and reverse transcribed to cDNA using oligo(dT) primer (RevertAid First Strand cDNA Synthesis Kit; Fermentas). RT-PCR for simultaneous detection and quantification of the cDNA samples was performed on an ABI StepOnePlus™ Real-Time PCR System and analysed with the StepOne software (Applied Biosystems, Life Technologies Corp., Carlsbad, CA, USA). Fifty nanograms of cDNA sample was used in a SYBR Green-based qPCR reaction; the cycling conditions were as follows: 50°C for 2 min., 95°C for 10 min., followed by 40 cycles of 95°C for 10 sec. and 60°C for 1 min. The end-point used in the real-time quantification was calculated by the StepOne software, and the threshold cycle number (Ct value) for each analysed sample was calculated. Each target gene was normalized to GAPDH to derive the change in Ct value (ΔCt). The primer sequences used in this study were as follows: *Col1a1*: 5′-GGGTGACCGTGGTGAGA-3′ and 5′-CCAGGAGAGCCAGAGGTCC-3′; *Col1a2*: 5′-TCCAAGGACAAGAAACAC-3′ and 5′-GCAGCCATCTACAAGAAC-3′; *Acta2*: 5′-AGCACATGGAAAAGATCTGGCACC-3′ and 5-TTTTCTCCCGGTTGGCCTTG-3′; *Zeb1*: 5′-AGCAGTGAAAGAGAAGGGAATGC-3′ and 5′-GGTCCTCTTCAGGTGCCTCAG-3′; *Gapdh*: 5′-ACCACAGTCCATGCCATCAC-3′ and 5′-TCCACCACCCTGTTGCTGTA-3′.

### Statistical analysis

Quantitative data were presented as the mean ± SD. The comparisons between two groups were analysed with Student's *t*-test. The comparisons among multiple groups (more than two) were analysed with repeated measure anova followed by Tukey-Kramer's post hoc test to identify differences among specific groups. For immunohistochemistry analysis, Fisher's exact test was used. A *P* value of less that 0.05 was considered significantly different.

## Results

### ZEB1 is significantly up-regulated in OSF

**Figure 1:**
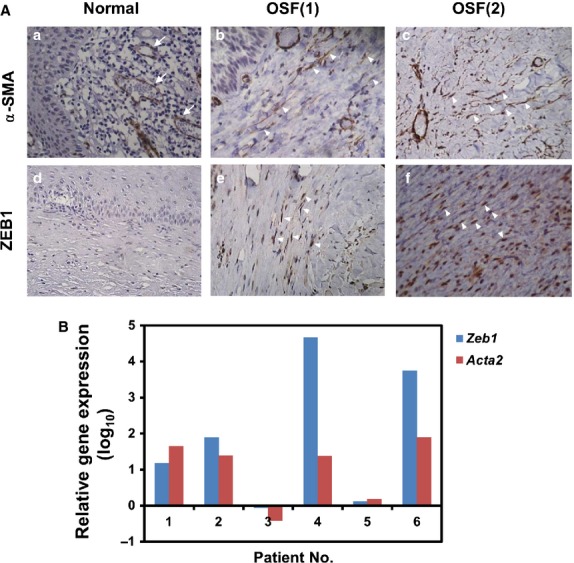
Zinc finger E-box binding homeobox 1 (ZEB1) is up-regulated in oral submucous fibrosis (OSF) tissues. (A) Paraffin sections of normal buccal mucosa (a, d) or OSF tissues (b, c, e, f) were obtained, and the expression of α-smooth muscle actin (α-SMA; a–c) or ZEB1 (d–e) was identified by immunohistochemistry (400×) as described in the Materials and Methods section. Arrows indicated positive vessels with α-SMA (a). Arrowheads indicated positive fibroblasts with α-SMA (b, c) or nuclear ZEB1 (e, f). (B) RNA were extracted from six paired normal/OSF mucosa tissues and used for quantification of *Zeb1* and *Acta2* expression by quantitative RT-PCR. Data were presented as relative gene expression in OSF mucosa tissues in comparison with their normal counterparts.

By immunohistochemistry, we examined the expression of α-SMA, a marker of myofibroblasts, and ZEB1 in OSF tissues obtained from patients with an areca quid chewing habit (Fig. 1A). α-SMA was expressed in the blood vessels of normal buccal mucosa (Fig. 1Aa), and α-SMA was found in both blood vessels and fibroblasts, which were identified as spindle shaped, in OSF cases (Fig. 1Ab and Ac). Zinc finger E-box binding homeobox 1 staining in normal buccal mucosa was weak positive (Fig. 1Ad), and nuclear ZEB1 was found in fibroblasts in OSF tissues (Fig. 1Ae and Af). In the 30 OSF cases examined, 80% or 73.3% of cases displayed strong expression of ZEB1 or α-SMA respectively (*P* < 0.05 when compared with normal mucosal tissues, Table 1). In contrast, only 30% of normal cases showed strong expression of ZEB1 or α-SMA. We further enrolled six paired samples of normal/OSF tissues to investigate the mRNA expression of ZEB1 (*Zeb1*) and α-SMA (*Acta2*; Fig. 1B). With quantitative RT-PCR analysis, the evaluated transcription of *Zeb1* and *Acta2* in OSF tissues when compared with their normal counterparts was observed in 83.3% of cases (Fig. 1B). These data reveal that ZEB1 expression is up-regulated in OSF tissues at both protein and transcriptional level.

**Table 1:** ZEB1 and α-SMA expression in human oral submucous fibrosis

	Case Number	Weak/moderate	Strong	*P* value
ZEB1				
Normal	10	7	3	
OSF	30	6	24	0.006
α-SMA				
Normal	10	7	3	
OSF	30	8	22	0.024

ZEB1 or α-SMA expression in human normal buccal mucosa or OSF was determined by immunohistochemistry as described in Materials and methods section. Specimens with 10–50% immunoreactive cells were considered as weak/moderate expression and there were more than 50% immunoreactive cells, which were defined as strong expression. *P* value was calculated by Fisher's exact test.

### ZEB1, as well as myofibroblast activity, is induced by transient treatment of BMFs with arecoline

**Figure 2:**
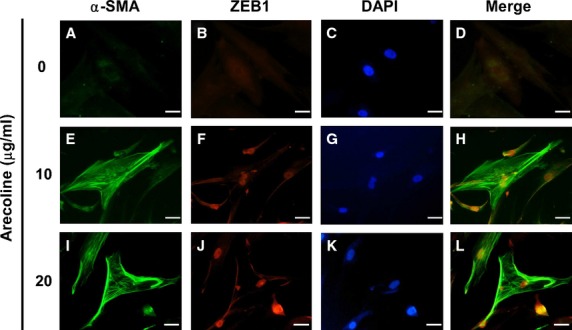
Arecoline induces the expression of α-smooth muscle actin (α-SMA) and Zinc finger E-box binding homeobox 1 (ZEB1) in buccal mucosal fibroblasts (BMFs). BMFs were serum starved (0.5% fetal bovine serum) for 48 hrs and treated with the indicated concentration of arecoline for a further 24 hrs in serum-free medium. The expression of α-SMA (**A, E, I**) or ZEB1 (**B, F, J**) was detected by immunofluorescence staining as described in Materials and Methods. DAPI (**C, G, K**) was used as a counterstain; scale bar = 10 μm. The experiments were repeated for three times and data from one experiment were presented.

**Figure 3:**
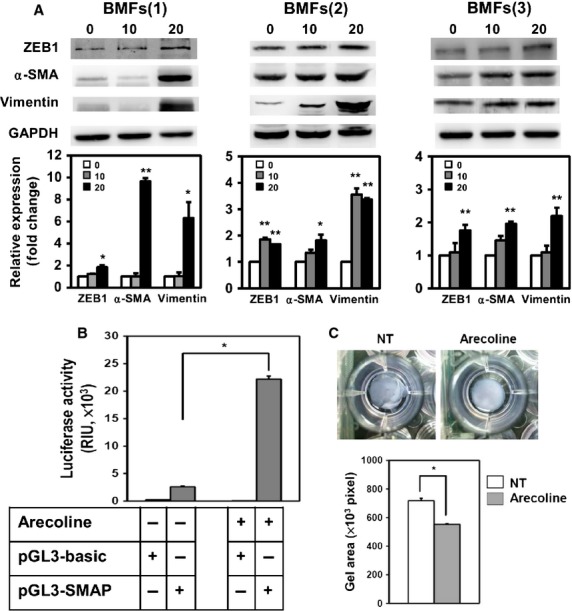
Arecoline induces the transdifferentiation of buccal mucosal fibroblasts (BMFs) into myofibroblasts. (A) Two lines of patient-derived BMFs were serum starved (0.5% FBS) for 48 hrs and treated with the indicated concentration of arecoline (μg/ml) for a further 24 hrs in serum-free medium. The expression of α-smooth muscle actin (α-SMA), vimentin, zinc finger E-box binding homeobox 1 or twist1 was detected by western blotting. GAPDH was used as a protein loading control. The relative expression of the target proteins to the control (0 μg/ml) was calculated from two independent experiments. *P < 0.05; **P < 0.01. (B) BMFs were transfected with control (pGL3-basic) or α-SMA promoter reporter (pGL3-SMAP) vectors for 24 hrs and treated with 20 μg/ml arecoline for a further 24 hrs. The luciferase activity was determined as described in Materials and Methods. The experiments were repeated three times and data from one experiment were presented. (C) BMFs were embedded into a collagen gel without (NT) or with 20 μg/ml arecoline and cultured for 48 hrs. The gel areas were calculated from two independent experiments using ImageJ software. *P < 0.05.

Arecoline is the major areca nut alkaloid and has been suggested to contribute the pathogenesis of OSF. We next examined if arecoline could induce myofibroblast transdifferentiation from human primary BMFs. As shown in Figure 2, we first treated BMFs with arecoline and used immunofluorescence analysis to observe the expression of α-SMA, the marker of myofibroblasts. With treatment of 10 or 20 μg/ml arecoline, α-SMA positive stress fibres were up-regulated in BMFs (Fig. 2e and i). We also observed the expression of nuclear ZEB1 in BMFs with α-SMA positive stress fibres after arecoline treatment (Fig. 2f and j). We next used western blotting to quantify the protein expression of α-SMA, vimentin and ZEB1 in arecoline-treated BMFs. The treatment of two BMF strains with 20 μg/ml arecoline was found to induce α-SMA expression, as well as vimentin expression, which is an intermediate filament protein to be overexpressed in OSF tissues 16 (Fig. 3A). The expression of ZEB1 was also induced in BMFs with increased α-SMA expression (Fig. 3A). By quantitative RT-PCR, we found that arecoline could up-regulate the transcription of ZEB1 (Fig. S1). Using a luciferase-based reporter assay, arecoline treatment activated SMA promoter activity (Fig. 3B). In addition to α-SMA expression, arecoline also caused the contraction of BMFs embedded into a collagen gel (Fig. 3C). These results suggest that arecoline could induce myofibroblast transdifferentiation in BMFs by up-regulating ZEB1.

### ZEB1 mediates arecoline-induced α-SMA expression in BMFs

**Figure 4:**
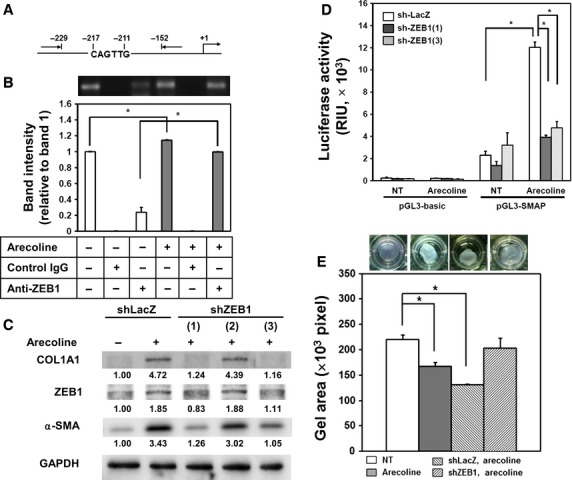
Arecoline-induced α-smooth muscle actin (α-SMA) expression is mediated by the direct binding of zinc finger E-box binding homeobox 1 (ZEB1) to the α-SMA promoter. (A) An illustration of the region containing the E-box domain in the α-SMA promoter. (B) The binding of ZEB1 to the E-box region in the α-SMA promoter was determined by a ChIP assay as described in Materials and Methods. Lane 1 and lane 4 represented input chromatins from non-treated or arecoline-treated cells respectively. Lane 2 and land 5 represented control IgG-precipitated chromatins from non-treated or arecoline-treated cells respectively. Land 3 and land 6 represented anti-ZEB1 antibody precipitated chromatins from non-treated or arecoline-treated cells respectively. The intensities of each band were compared to land 1. Data were calculated from two independent experiments. *P < 0.05. (C) Buccal mucosal fibroblasts (BMFs) were transduced with the indicated shRNA lentiviruses for 4 days, and the expression of COL1A1, α-SMA or ZEB1 was determined by western blotting. GAPDH was used as a protein loading control. The inserted numbers indicate the relative expression of the target proteins to the control (shLacZ). (D) BMFs were transduced with the indicated shRNA lentivirus for 2 days and harvested for transfection with the control (pGL3-basic) or α-SMA promoter reporter (pGL3-SMAP) vectors. After treatment without (NT) or with arecoline (20 μg/ml) for 24 hrs, the luciferase activity was determined and normalized to RLuc activity. *P < 0.05 when compared with NT (shLacZ); #*P* < 0.05 when compared with arecoline-treated (shLacZ). (E) BMFs were transduced with shRNA lentivirus for 48 hrs and embedded into collagen gels with or without arecoline (20 μg/ml). After 48 hrs, contraction of the gels was photographed (upper panel), and the gel areas were calculated from two independent experiments using ImageJ software (lower panel). *P < 0.05.

To further understand the role of ZEB1 in the arecoline-induced α-SMA expression, we first noticed that there was an E-box region in the promoter region of α-SMA (-CAGTTG-, −217 to −211, Fig. 4A) according to the report of Blank *et al*. 17. We next examined if ZEB1 could bind to this E-box region in the α-SMA promoter. By chromatin immunoprecipitation, we found that arecoline treatment of BMFs increased the binding of ZEB1 to the α-SMA promoter (Fig. 4B). To further demonstrate that ZEB1 may mediate arecoline-induced α-SMA expression in BMFs, we used lentiviral-based shRNA to knockdown the expression of ZEB1. Zinc finger E-box binding homeobox 1 expression was decreased by two of three selected shRNA clones (shZEB1(1) and shZEB1(3)), and the increased α-SMA or COL1A1 expression caused by arecoline treatment was suppressed in cells transduced with these two shRNAs (Fig. 4C). We also examined α-SMA promoter activity in ZEB1 knockdown BMFs. The arecoline-induced α-SMA promoter activity was suppressed in ZEB1 knockdown BMFs (shZEB1(1) or shZEB1(3); Fig. 4D). In addition to α-SMA expression, knockdown of ZEB1 with pooled shRNAs suppressed the arecoline-induced collagen contraction of BMFs (Fig. 4E). Our data demonstrated that arecoline transiently induced ZEB1 expression in BMFs to induce α-SMA expression and promote BMFs to transdifferentiate into myofibroblasts.

### Long-term exposure of arecoline induces the expression of fibrogenic genes and is correlated with ZEB1 expression

**Figure 5:**
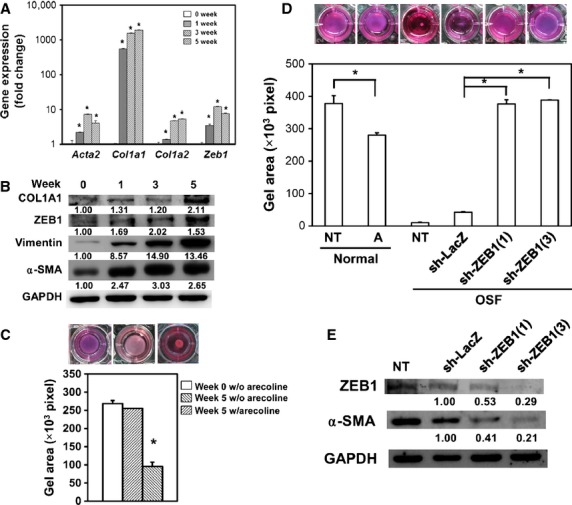
Long-term exposure of buccal mucosal fibroblasts (BMFs) to arecoline induces fibrogenic gene expression and collagen contraction. (A–C) BMFs were cultured with 10 μg/ml arecoline for 5 weeks. RNA was extracted weekly and reverse transcribed into cDNA. The expression of fibrogenic genes was determined using SYBR Green-based real-time PCR. *P < 0.05 (A). Cell lysates were collected weekly, and the expression of fibrogenic proteins was determined by Western blot. The inserted numbers indicate the relative expression of target proteins to expression at week 0 (B). Cells were embedded into a collagen gel and performed collagen contraction assay. Pictures of wells were taken at 72 hrs and the degree of gel contraction was quantified by measuring the gel area. *P < 0.05 (C). (D) BMFs from normal or fibrotic tissues of oral cavity of an oral submucous fibrosis patient were used for collagen contraction assay to access their myofibroblast activity. A, 10 μg/ml of arecoline; NT, no treatment. Data were presented from two independent experiments. *P < 0.05. (E) The expression of zinc finger E-box binding homeobox 1 (ZEB1) or α-smooth muscle actin in fibrotic BMFs after lentivirus-mediated ZEB1 knockdown was determined by western blot. Data were presented from one of two independent experiments. The inserted numbers represented the relative expression level of target protein when compared with sh-LacZ control lentivirus.

Areca quid chewing–associated OSF is a chronic process, and we examined the effect of long-term arecoline exposure on BMFs. We first confirmed that there was no obvious change in the expression of α-SMA (*Acta2*) or profibrogenic extracellular matrix genes (type I collagen *Col1a1* and *Col1a2*) as well as ZEB1, while cultivating BMFs without arecoline exposure for 4 weeks both in mRNA or in protein level (Fig. S2). When BMFs were exposed to 10 μg/ml arecoline, the mRNA level of *Acta2*,*Col1a1* or *Col1a2* was significantly and greatly up-regulated in a time-dependent manner (Fig. 5A). *Zeb1* mRNA expression positively correlated with the expression of fibrogenic genes (Fig. 5A). Protein expression of COL1A1, α-SMA, vimentin and ZEB1 in BMFs after long-term exposure to arecoline also displayed a similar pattern (Fig. 5B). We further found that the myofibroblast activity was induced in BMFs after long-term exposure of arecoline by collagen contraction assay (Fig. 5C).

### Silencing of ZEB1 suppressed the expression of SMA and myofibroblast activity of fibrotic BMFs isolated from an OSF patient with an areca quid chewing habit

We next used BMFs, which were isolated from fibrotic oral cavity tissues of an OSF patient with an areca quid chewing habit to evaluate the role of ZEB1 in their myofibroblast activity. By collagen contraction assay, BMFs from fibrotic oral cavity tissues displayed a myofibroblast phenotype (Fig. 5D). There was no myofibroblast activity of BMFs from normal oral cavity tissues of the same patient (Fig. 5E). With lentiviral delivery of specific shRNAs, silencing of ZEB1 decreased the expression of α-SMA (Fig. 5E), as well as collagen contraction activity (Fig. 5D) of these fibrotic BMFs. These data suggest that areca quid chewing–associated OSF could result from arecoline-induced ZEB1 expression in BMFs.

### Arecoline-induced ZEB1 expression was mediated by the activation of IGF-1R

**Figure 6:**
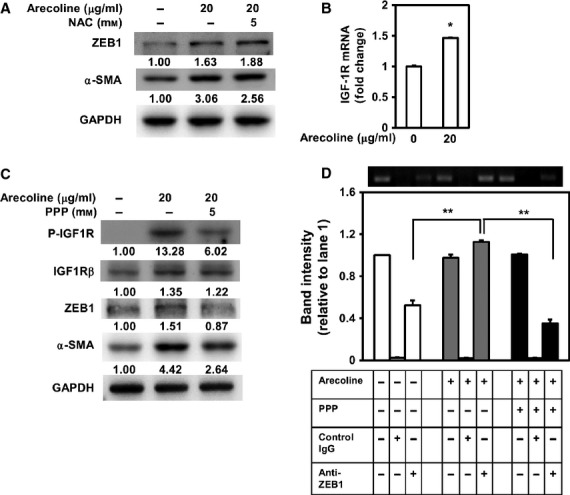
Arecoline-induced zinc finger E-box binding homeobox 1 (ZEB1) expression is independent of reactive oxygen species, but is mediated by the activation of insulin-like growth factor receptor-1 (IGF-1R). (A) Buccal mucosal fibroblasts (BMFs) were treated with 20 μg/ml of arecoline with or without *N*-acetyl cysteine treatment (5 mM) for 24 hrs and cells were harvested for analysis of the expression of ZEB1 and α-smooth muscle actin (α-SMA) by western blot. The inserted numbers represented the relative expression level of target protein when compared with BMFs without arecoline treatment. (B) BMFs were treated with or without 20 μg/ml of arecoline for 24 hrs and RNA were extracted for detection of mRNA level of IGF-1R by qRT-PCR method. Data were presented from two independent experiments. *P < 0.05. (C) BMFs were treated with 20 μg/ml of arecoline with or without picropodophyllin treatment (5 μM) for 24 hrs and cells were harvested for ChIP analysis to determine the binding of ZEB1 to E-box domain of α-SMA promoter. The intensities of each band were compared to the input of non-treated cells (band 1). Data were calculated from two independent experiments. *P < 0.05.

We next tried to figure out how arecoline induce the activation of ZEB1 in BMFs. We previously demonstrated that arecoline could induce oxidative responses in oral squamous carcinoma cells 18,19. We first used NAC, a scavenger of reactive oxygen species (ROS), to examine if ROS plays any role in arecoline-induced ZEB1 expression. When co-treated with 5 mM of NAC, it did not change the protein level of ZEB1 in arecoline-treated BMFs (Fig. 6A). It suggests that arecoline-induced ZEB1 expression was independent of ROS production. We previously have demonstrated that arecoline could up-regulate IGF-1 expression 8. We next investigated if IGF-1R signalling plays a role in arecoline-induced ZEB1 activation in BMFs. We first found that arecoline treatment up-regulated the transcription of IGF-1R mRNA (Fig. 6B). Arecoline also induced the phosphorylation of IGF-1R^Tyr1161^ in BMFs (Fig. 6B). When co-treated with 5 μM of PPP, a specific inhibitor of IGF-1R, the arecoline-induced phosphorylation of IGF-1R^Tyr1161^ as well as the expression of ZEB1 and α-SMA was decreased (Fig. 6B). The arecoline-induced binding of ZEB1 in α-SMA promoter could also be significantly suppressed by PPP treatment (Fig. 6C). These data suggest that arecoline-induced ZEB1 activation in BMFs is mediated by the activation of IGF-1R.

## Discussion

In this study, we attempted to investigate the role of ZEB1 in the pathogenesis of areca quid chewing–associated OSF. From OSF tissues, we discovered that ZEB1 was up-regulated in the nucleus of fibroblasts in OSF tissues (Fig. 1), and this result suggested that ZEB1 might be activated. We further discovered that arecoline could induce ZEB1 expression in BMFs and then up-regulate the expression of α-SMA and cause myofibroblast transdifferentiation. Myofibroblasts are the contractile fibroblasts that contribute to tissue repair during wound healing 6, but myofibroblasts have a pathological role in tissue fibrosis by causing dysregulated contraction and secretion of ECM proteins. We previously found that arecoline acted not only as an inhibitor on the gelatinolytic activity of metalloproteinase (MMP)-2 but also a stimulator for tissue inhibitor of metalloproteinase-1 (TIMP-1) activity in BMFs, which might account for the excessive accumulation of ECM proteins in OSF 20. Recently, the overexpression of TIMP-1 in canine kidney epithelial MDCK cells was found to induce the expression of ZEB1 and lead to an EMT-like process that was independent of its MMP-inhibitory domain 21. Whether the induction of ZEB1 in BMFs by arecoline is also mediated by TIMP-1 is unknown. We have recently demonstrated that arecoline could induce the expression of S100A4 to mediate the activity of collagen contraction of BMFs 22. By promoter analysis, we also noticed that there were several potential E-box motifs within the promoter region of S100A4 (Lai YL, Chang YC, Yu CC, Chang WW, unpublished observation). It will be interesting to investigate if ZEB1 mediates the expression of S100A4 under arecoline stimulation.

A number of cytokines have been demonstrated to be profibrotic cytokines, including TGF-β, platelet-derived growth factor and IGF-1 23. Transforming growth factor-β is a multifunction cytokine with a well-known pathological role in organ fibrosis. Recently, activation of TGF-β1 in arecoline-treated oral keratinocytes in an integrin αvβ6–dependent manner was shown to induce the transdifferentiation of human oral fibroblasts into myofibroblasts 4. In vascular diseases, TGF-β–mediated differentiation of vascular smooth muscle cells (SMCs) has been suggested to play a pathological role and to be mediated by ZEB1 expression 24. Transforming growth factor-β–induced ZEB1 in SMCs could interact with Smad3 and SRF to induce the transcription of α-SMA. It suggests that ZEB1 is critical in TGF-β-mediated differentiation of SMCs 24. Here, we directly treated BMFs with arecoline to induce myofibroblast transdifferentiation and provided a similar mechanism that the up-regulation of α-SMA by arecoline is mediated by direct binding of ZEB1 to its promoter. Recently, areca nut extracts, as well as arecoline, could induce the activation of TGF-β signalling in human keratinocyte HaCaT cells 25. Areca nut extracts could also induce the activation of TGF-β signalling and the expression of α-SMA and COL1A1 in human gingival fibroblasts 25. In the view that TGF-β could induce the expression of ZEB1 in various cell types 26, it is possible that areca nut extracts or arecoline-induced α-SMA expression in BMFs could be mediated by TGF-β signalling and remains to be further investigated.

Although we previously reported that arecoline could induce oxidative-stress responsive proteins in oral squamous cell carcinomas, such as haem oxygenase-1 18 and metallothionein-1 19 and it could be inhibited by NAC treatment, but here we found that arecoline-induced ZEB1 expression in BMFs was independent of ROS induction (Fig. 6A). It is consistent with the findings from a recent study, which demonstrated that NAC or catalase treatment could not inhibit the collagen contraction activity of BMFs by treatment of areca nut extracts 27. In a breast cancer study, IGF-1 could up-regulate ZEB1 to promote the invasion of the metastatic breast cancer cell line MDA-MB-231 28. Previously, we found that arecoline could induce the expression of IGF-1 in BMFs 8. Here, we further demonstrate that arecoline-induced ZEB1 expression is mediated by the activation of IGF-1R. In carbon tetrachloride–induced rat hepatic fibrosis model, the anti-fibrotic effect of (-)-epigallocatechin-3-gallate (EGCG) has been demonstrated to be associated with its effect in down-regulation of IGF-1R expression 29. Blockade of IGF-1R activation in bleomycin-induced mouse lung fibrosis also displayed activities in induction of fibroblast apoptosis and resolution of pulmonary fibrosis 30. Together with our data, it brings a new insight to develop therapeutic strategies in OSF by IGF-1R inhibitors or neutralization antibodies.

Currently, no effective drug for OSF exists. Current management of OSF includes stopping the areca quid chewing habit, medication and surgical intervention. In early OSF without trismus, cessation of the areca quid chewing habit improves the disease 31. Because OSF is considered an inflammatory disease, anti-inflammation drugs, such as steroids or Levamisole, have been used in the treatment of OSF 32. Other modifications of OSF include dietary supplementation with iron and vitamins or injection of hyaluronidase or collagenase to facilitate the removal of fibrotic tissues 33. Here, we demonstrated the involvement of ZEB1 in areca quid–associated OSF. A screen to identify the potential therapeutic effects of natural compounds that have been reported to inhibit ZEB1 expression, such as resveratrol 34, EGCG 35 and sulforaphane 36, would be worthwhile.

**Figure 7:**
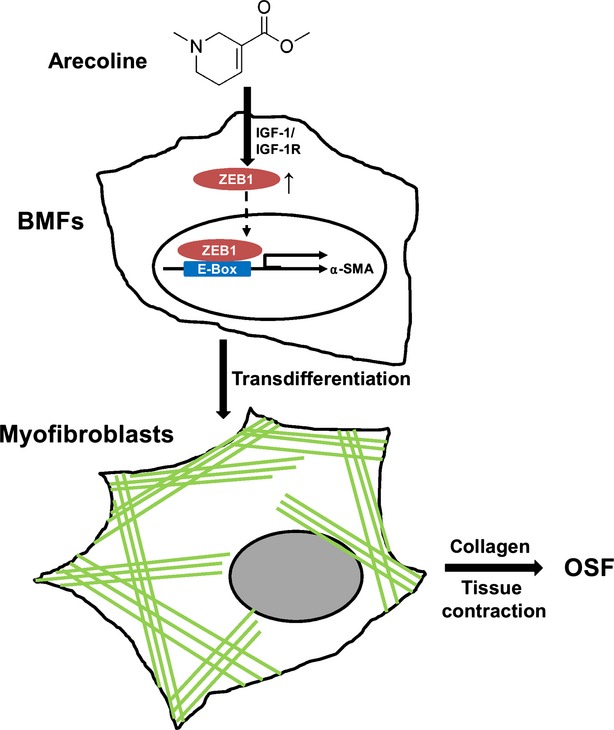
Working model for the role of zinc finger E-box binding homeobox 1 (ZEB1) in arecoline-induced oral submucous fibrosis (OSF). Our data provide evidence that arecoline-induced ZEB1 activation in buccal mucosal fibroblasts (BMFs) is mediated by insulin-like growth factor receptor-1 activation and BMFs transdifferentiate into myofibroblasts by ZEB1-driven α-smooth muscle actin expression. These myofibroblasts further lead to the accumulation of extracellular matrix in buccal mucosa, cause tissue contraction and finally progress into OSF.

In summary, we explored a possible role of ZEB1 in the pathogenesis of areca quid–associated OSF; this role has been suggested in Figure 7. We discovered that ZEB1 could be induced by arecoline in BMFs through the activation of IGF-1R. The arecoline-induced ZEB1 could bind to the E-box domain in the α-SMA promoter to up-regulate α-SMA expression and myofibroblast transdifferentiation from BMFs (Fig. 8). Long-term exposure of arecoline in BMFs also promoted fibrogenic gene expression. The up-regulation of ZEB1 was also observed in OSF tissues from patients with an areca quid chewing habit. These data provide new insights into the development of drugs for OSF by targeting ZEB1 expression.

## Supporting information

Figure S1 mRNA level of *Col1a1, Zeb1 and Acta2* in short-term arecoline-treated BMFs.

Figure S2 The expression of fibrogenic genes and ZEB1 in long-term cultured normal BMFs.
