# Supplementary figures and images for: Arecoline-induced myofibroblast transdifferentiation from human buccal mucosal fibroblasts is mediated by ZEB1
Source: J Cell Mol Med. 2014 Jan 8;18(4):698–708. doi: 10.1111/jcmm.12219 (PMC4000120; doi:10.1111/jcmm.12219)

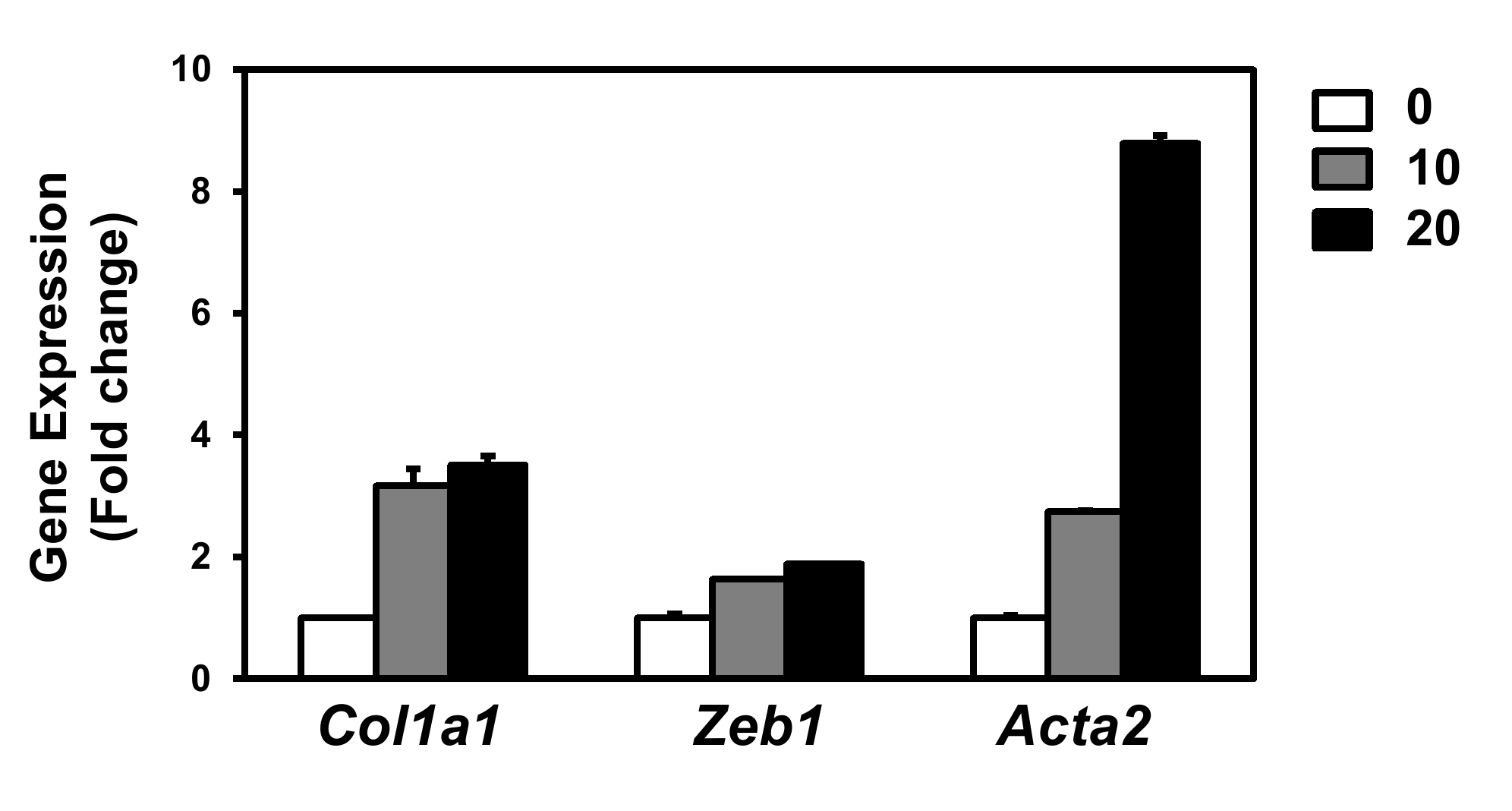

Supplement: Figure S1 [file jcmm0018-0698-sd1.tif]

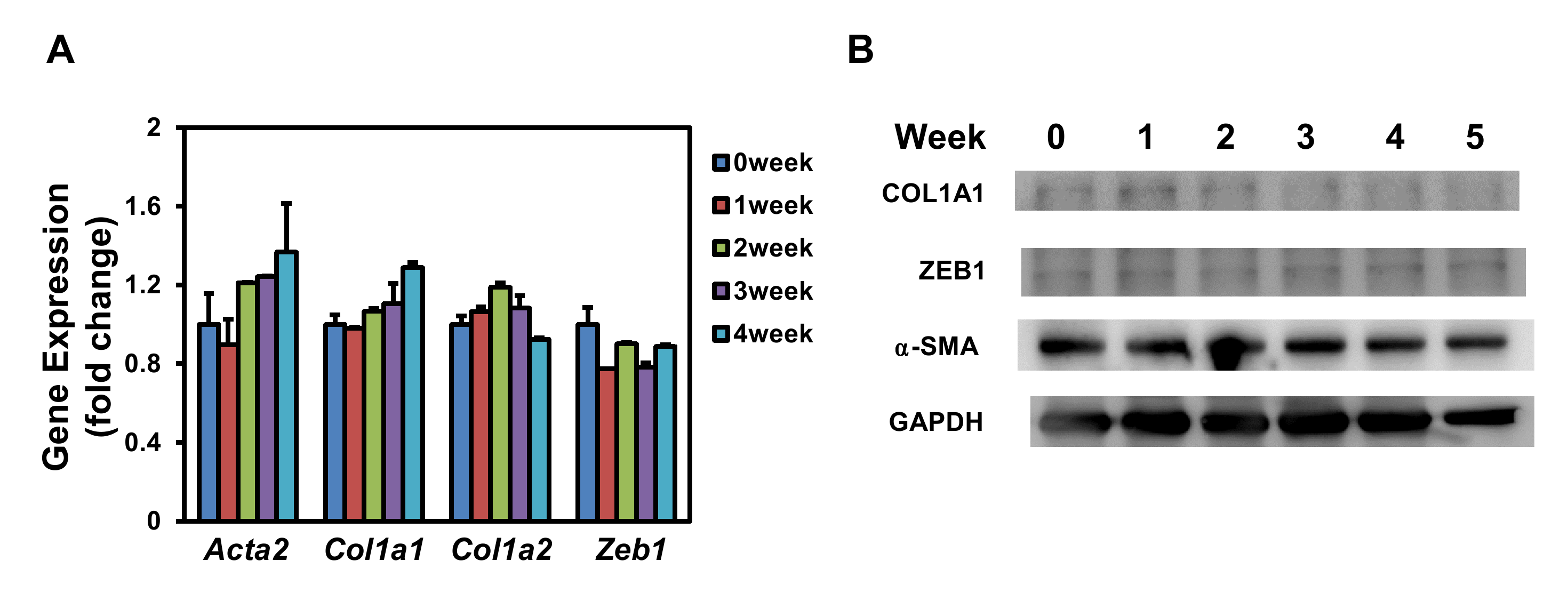

Supplement: Figure S2 [file jcmm0018-0698-sd2.tif]
