# Supplementary material for: Arecoline-induced myofibroblast transdifferentiation from human buccal mucosal fibroblasts is mediated by ZEB1
Source: J Cell Mol Med. 2014 Jan 8;18(4):698–708. doi: 10.1111/jcmm.12219 (PMC4000120; doi:10.1111/jcmm.12219)
Supplement: Supplementary file 3 [file jcmm0018-0698-sd3.docx]

Supporting information

**Figure S1. mRNA level of *Col1a1*, *Zeb1*, and *Acta2* in short-term arecoline treated BMFs.** RNA were extracted from BMFs with or without arecoline treatment for 24 h and mRNA level of *Col1a1*, *Zeb1* and *Acta*2 was determined by SYBR Green based quantitative RT-PCR. Data were presented as relative expression in comparison with BMFs without arecoline treatment from one of two independent experiments.

**Figure S2. The expression of fibrogenic genes and ZEB1 in long-term cultured normal BMFs.** RNA or proteins were extracted from BMFs weekly without arecoline treatment. The expression of fibrogenic genes or ZEB1 was determined by SYBR Green based quantitative RT-PCR (A) or western blot (B).
